# 

**Published:** 1892-12-31

**Authors:** 


					u price twopence.
W Vol. XIII.-
-Dec. 31st. 1892.
to**1. the General Pent Office a* a Xewipaper for
**??&iaBion ia the United Kingdom and Abroad.]
WTh"extra~RUE3ing supplement .
Per Aunnm, 8s. 6d., post free.
Monthly Parts, 6d.; post free, 8d.
Ditto per Annum, 8s.. post free.

				

